# Symmetry and Shannon Measure of Ordering: Paradoxes of Voronoi Tessellation

**DOI:** 10.3390/e21050452

**Published:** 2019-04-30

**Authors:** Edward Bormashenko, Irina Legchenkova, Mark Frenkel

**Affiliations:** Department of Chemical Engineering, Biotechnology and Materials, Engineering Sciences Faculty, Ariel University, Ariel 407000, Israel

**Keywords:** Voronoi entropy, Voronoi tessellation, symmetry, ordering, Shannon measure of information

## Abstract

The Voronoi entropy for random patterns and patterns demonstrating various elements of symmetry was calculated. The symmetric patterns were characterized by the values of the Voronoi entropy being very close to those inherent to random ones. This contradicts the idea that the Voronoi entropy quantifies the ordering of the seed points constituting the pattern. Extension of the Shannon-like formula embracing symmetric patterns is suggested. Analysis of Voronoi diagrams enables the elements of symmetry of the patterns to be revealed.

## 1. Introduction

Symmetry considerations play a key role in modern science [1,2,3], giving rise to the conservation laws in physics and being dominant in quantum theory [4], crystallography [5], condensed-matter physics [6], thermodynamics, chemistry, and biology [1,2,3]. Moreover, symmetry considerations are of fundamental importance in aesthetics and science–art relations [1,7,8]. The other fundamental value playing a central role in modern science is the Shannon measure of information [9,10,11,12,13,14]. In the present article, we demonstrate that the analysis of the Voronoi diagrams (or Voronoi tessellations) enables the synthesis of symmetry and the Shannon measure of ordering considerations [15,16,17,18]. Voronoi diagrams arise from problems involving patterns with a surface distribution of spots [19]. A Voronoi tessellation (or diagram or mosaic) involves the partitioning of a plane into regions based on the distance to a specified discrete set of points (called seeds, sites, nuclei, or generators) [17,18]. For each seed, there is a corresponding region consisting of all points closer to that seed than to any other. The Voronoi polyhedron of a point nucleus in space is the smallest polyhedron formed by perpendicularly bisecting planes between a given nucleus and all the other nuclei. The Voronoi tessellation divides a region into space-filling, non-overlapping convex polyhedrons [17,18,19,20]. The idea of what is now called the Voronoi tessellation had already been proposed by Johannes Kepler and Rene Descartes in the 17th century [16,18,21]. Kepler used it to study the densest sphere packing problem, whereas Descartes employed these tessellations to verify that the distribution of matter in the Universe forms vortices centered at fixed stars [21].

It is generally accepted that the Voronoi diagrams allow the orderliness of the 2D distribution of points to be quantified with the so-called Voronoi entropy, defined according to the Shannon-like formula as:

(1) $$S_{vor}=\sum_{i}P_{i}lnP_{i}$$

where *P_i_* is the fraction of polygons with *k* sides or edges (also called the coordination number of the polygon) in a given Voronoi diagram [10,11,12]. The summation in Equation (1) is performed from *i* = 1 (corresponding to *k* = 3) to the largest coordination number of any available polygon, e.g., to *i* = 4 (corresponding to *k* = 6) if a polygon with the largest number of edges is a hexagon. In our recent paper, we demonstrated that the labeling of the value *S_vor_* with the wording “Voronoi entropy” was misleading and confusing [22]. In actuality, Equation (1) quantifies the average Shannon measure of ordering of a given 2D pattern [9,10,11,12,13,14,22]. In the present article, we show that the situation becomes even more complicated when the given pattern demonstrates elements of symmetry (centers, axes). In this case, the Shannon measure of ordering calls for redefinition.

## 2. Results and Discussion

### 2.1. 2D Patterns Demonstrating Symmetry and Their Voronoi Tessellations

**Figure 1:**
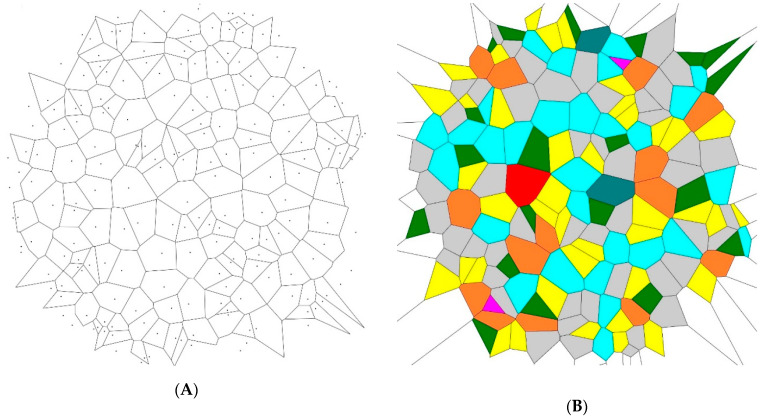
The Voronoi tessellation for the set of 200 random points is depicted. The Voronoi entropy is *S_vo_*_r_ = 1.65. (**A**) The initial set of points generating the Voronoi tessellation. (**B**) Colored Voronoi polygons.

We analyzed Voronoi diagrams built for sets of points demonstrating different elements of symmetry (namely centers and axes of symmetry, as well as rotational symmetry). In the first stage, the random sets of 200 and 1000 points located inside a circle of a given diameter were generated by the MATLAB software script. The Voronoi tessellation for the set of 200 random points is depicted in Figure 1. The Voronoi entropy was calculated with the moduli of the software developed at the Department of Physics and Astronomy at the University of California (Department of Physics and Astronomy University of California, Irvine) (https://www.physics.uci.edu/~foams/do_all.html). The procedure was repeated 20 times, and the average value of the Voronoi entropy and its standard deviation was established as $S_{vor}=1.66\pm 0.05$ for the set of 200 and as $S_{vor}=1.68\pm 0.02$ for the set of 1000 points (seeds). This value was close to the Voronoi entropy $S_{vor}=1.71$ reported for random point patterns by other groups [23,24]. It is reasonable to attribute the difference between our calculations and the reported results to the boundary effects occurring at the circumference of the pattern (see Figure 1). These random patterns are herein called “initial patterns”.

**Figure 2:**
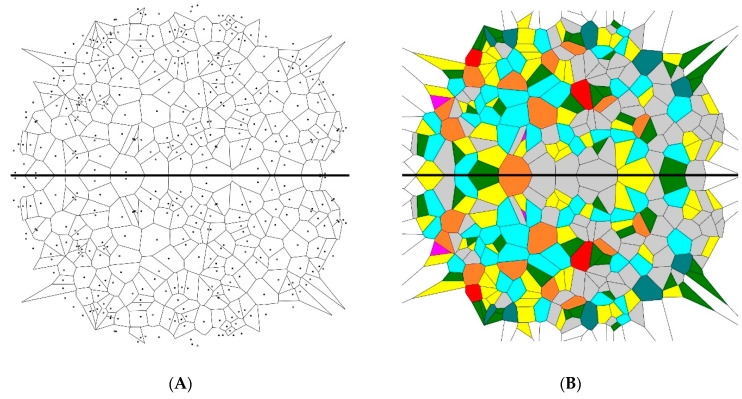
The mirror image of the Voronoi diagram depicted in Figure 1 is shown. The value of the Voronoi entropy is *S_vor_* = 1.68. (**A**) Set of points generating the Voronoi construction. (**B**) Colored Voronoi polygons.

In the second stage, the mirror images of the 20 initial patterns were built and placed on the initial patterns, as shown in Figure 2. Then, their Voronoi entropy was calculated (the number of points in the new diagrams was twice that of the initial ones, while the area remained the same). The average value of the Voronoi entropy was established as $S_{vor}=1.64\pm 0.05$ for a set of 200 and as $S_{vor}=1.68\pm 0.01$ for a set of 1000 seed points. The difference between the values of the Voronoi entropy of the initial and “mirror reflected” images was within the statistical uncertainty of the calculation. Thus, we concluded that the mirror reflection of the initial patterns did not change their Voronoi entropy.

**Figure 3:**
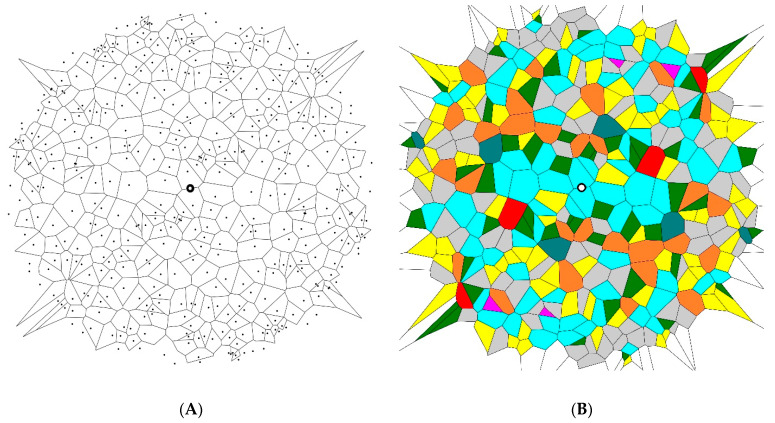
The point symmetry (inverse) image of the pattern depicted in Figure 1 is shown. The value of the Voronoi entropy is *S_vor_* = 1.69. (**A**) Set of points generating the Voronoi tessellation. (**B**) Colored Voronoi polygons.

In the third stage, the point symmetry (inverse) images of the 20 initial patterns were built and put on the initial patterns, and their Voronoi entropy was calculated (again, the entire number of points in the new Voronoi mosaics was twice that of the initial ones). The average value of the Voronoi entropy inherent to patterns similar to those depicted in Figure 3 was established as $S_{vor}=1.66\pm 0.06$ for a set of 200 and as $S_{vor}=1.67\pm 0.02$ for a set of 1000 points.

**Figure 4:**
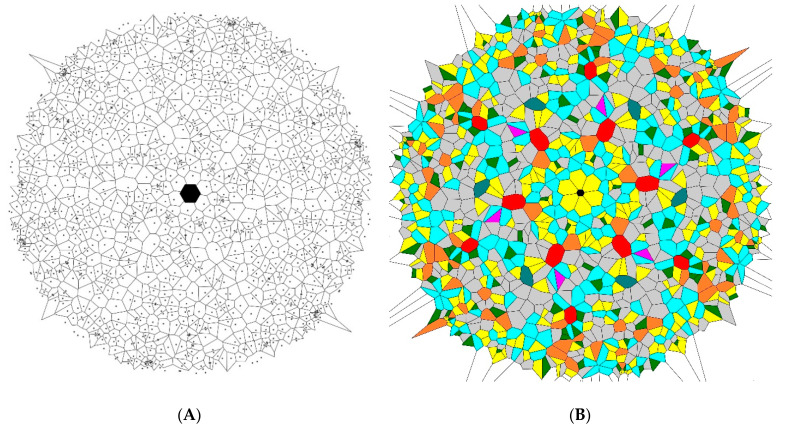
The six-fold symmetry pattern obtained by the rotation of the initial pattern shown in Figure 1 is depicted. The value of the Voronoi entropy is *S_vor_* = 1.57. (**A**) Set of points generating the Voronoi tessellation. (**B**) Colored Voronoi polygons.

In the fourth stage, the initial images were rotated for the angles $\frac{\pi }{3},\;\frac{2\pi }{3},\;\pi ,\;\frac{4\pi }{3},\;\mathrm{and}\;\frac{5\pi }{3}$ rad, as shown in Figure 4, and the pattern built of 1200 points and demonstrating the six-fold symmetry was created. The average value of the Voronoi entropy of these mosaics was established as $S_{vor}=1.65\pm 0.07$ for a set of 200 and as $S_{vor}=1.67\pm 0.02$ for a set of 1000 points.

**Figure 5:**
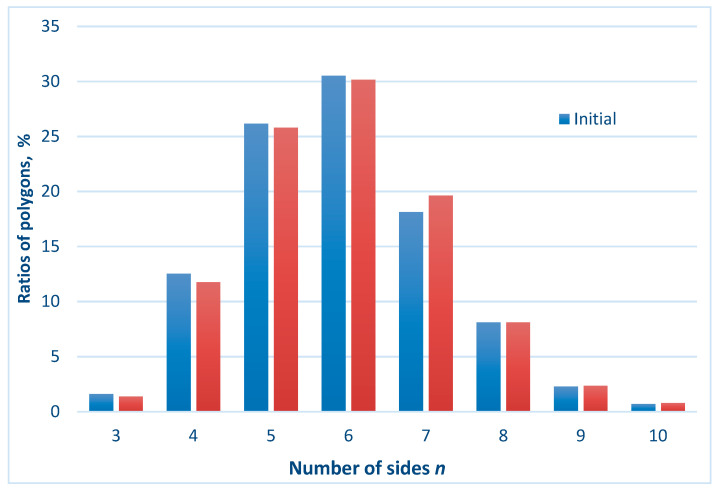
The ratios of different kinds of polygons in the initial (random) and inverse patterns are shown.

Hence, we conclude that the studied symmetry transformations did not change the Voronoi value of the patterns. This was true for the inversion, mirror reflection, and rotational symmetry operations. This result was quite trivial, as summarized in Table 1. Indeed, the ratio of *n*-polygons in the initial patterns and in the transformed ones remained the same. Thus, the value of the Voronoi entropy, calculated according to Equation (1), was also expected to be the same, as demonstrated in Figure 5. However, the patterns depicted in Figure 2, Figure 3 and Figure 4 definitely appeared much more “ordered” than that of the initial random pattern shown in Figure 1. If we adopt the notion that the Voronoi entropy is a true measure of ordering 2D patterns, its value established for symmetric, “ordered” patterns, depicted in Figure 2, Figure 3 and Figure 4, would be expected to be much lower than the Voronoi entropy of the initial random mosaic shown in Figure 1. The paradox could not be resolved by the suggestion that the symmetric mosaics shown in Figure 2, Figure 3 and Figure 4 contain a larger number of points. Recall that the Voronoi entropy is the intensive parameter of the regular pattern, and it is independent of the number of points or their density (when the “boundary effects” were neglected). For example, the Voronoi entropy of the pattern built only of squares (or other identical polygons) was equal to zero, whatever the number (or density) of points constituting the pattern. The Voronoi entropy of the random pattern tended to be 1.71, whatever the number/density of seed points.

The Voronoi entropy did not change dramatically under the symmetry transformations because almost all of the random patterns already appeared over the initial 2D distribution of points shown in Figure 1. Mirroring or other symmetry operations which did not change the ratio of polygons would keep the entropy almost constant. This was not exactly constant due to the “boundary effects”.

In order to resolve the puzzle and to quantify ordering appearing in various patterns, we propose to redefine the generalized Voronoi entropy for all kinds of patterns (symmetric and non-symmetric ones) as follows:

(2) $$S_{vor}^{gen}=\frac{S_{vor}}{N}=\frac{S_{vor}}{n+1}$$

where *S_vor_* is calculated according to Equation (1), *N* is the total number of the symmetry elements inherent to the pattern, including the trivial symmetry operation (in other words the rotation of the pattern to 2π rad), and *n* is the number of the non-trivial symmetry operations inherent to the pattern. Equation (2) contains Equation (1) as a particular case. Indeed when *N* = 1 and *n* = 0, the 2D pattern has the trivial axis of symmetry only and we return to Equation (1), namely: $S_{vor}^{gen}=S_{vor}=\sum_{i}P_{i}lnP_{i}$. However, for the symmetric patterns, such as those depicted in Figure 2, Figure 3 and Figure 4, the generalized Voronoi entropy supplied by Equation (2) is half (*N* = 2) of that calculated for the random mosaics shown in Figure 1. Hence, the suggested Equation (2) better quantifies the “ordering” of 2D patterns than the “standard” Equation (1).

### 2.2. Revealing Symmetry in 2D Patterns with Voronoi Diagrams

Now consider the arbitrary 2D pattern containing *p* points and the following question: Does it demonstrate the elements of symmetry? We show that the Voronoi tessellation may help answer this question. In the first stage, we constructed the Voronoi diagram of the pattern. Edges and vortices of the diagram formed the simply connected graph. The number of edges *E* of the graph is given by the Euler formula:

(3) E=P+V−χ

where *P* is the number of Voronoi polygons, *V* is the number of the vertices of the graph (in other words of the Voronoi diagram), and χ=2 is the Euler characteristics of the surface. Now, we sort the edges of the graph according to their length *L.* The number of edges (say *n* of them) may have the same length *L*. Thus, the probability to find the edge with the length *L* is given by: $P_{L}=\frac{n\left(L\right)}{E}$. Let us define the Voronoi entropy of the graph as follows:

(4) $$S_{vor}^{graph}=-\sum_{i}P_{Li}lnP_{Li}.$$

**Figure 6:**
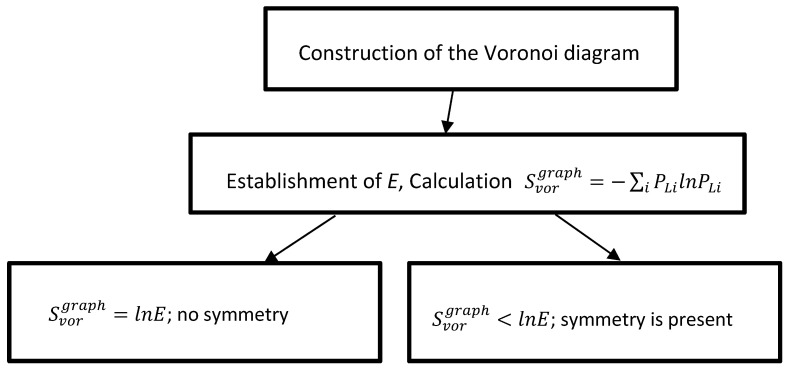
The algorithm enabling the finding of elements of symmetry for a given pattern of seed points.

If all of the edges constituting the graph are of different lengths, its Voronoi entropy equals $S_{vor}^{graph}=lnE$, meaning that the pattern demonstrates no symmetry. However, when the Voronoi diagram demonstrates the elements of symmetry, edges of the same length appear at the Voronoi tessellation, and the Voronoi entropy of the graph, given by Equation (4), are necessarily decreased. For example, if the Voronoi diagram has the mirror symmetry (in other words it has the axis of symmetry of the second order), the simple calculation according to Equation (4) yields: $S_{vor}^{graph}=ln\frac{E}{2}$. The formula is easily generalized for the single axis of symmetry with an order of *m*, namely: $S_{vor}^{graph}=ln\frac{E}{m};m=Eexp\left(-S_{vor}^{graph}\right)$. Thus, we can conclude that the Voronoi entropy of the graph is sensitive to the symmetry of the initial pattern, and enables the disclosure of the elements of symmetry of the pattern (or their absence), making possible the establishment of the number *N* appearing in Equation (2). The procedure enabling the finding of elements of symmetry is presented in Figure 6. However, consider that the suggested procedure does not enable the establishment of *N*, defined as the total number of the symmetry elements inherent to the pattern appearing in Equation (2).

## 3. Conclusions

It is generally agreed that the Shannon-shaped Voronoi entropy quantifies the ordering of 2D point patterns [17,25]. We have already mentioned that the labeling of the average Shannon measure of ordering by the notion of Voronoi entropy was confusing due to the fact that it had no relation to the thermodynamic entropy [22]. In the present paper, we demonstrated that involving the traditional Shannon-like formula for the characterization of ordering in symmetric patterns resulted in misleading estimations. It turns out that the Voronoi entropy of random patterns is equal to that of symmetric ones possessing centers or axes of symmetry, which are apparently more ordered than random mosaics. The traditional expression for the Voronoi entropy works well for non-symmetric patterns. In order to quantify the ordering inherent to all kind of patterns, we suggest an expression applicable to symmetrical and non-symmetrical point patterns. Analysis of the Voronoi tessellations enables the elements of symmetry of the patterns to be found.

## Figures and Tables

**Table 1:** Voronoi entropy with its standard deviation calculated for different sets of points.

Sample	Voronoi Entropy	Standard Deviation	Random Sample	Voronoi Entropy	Standard Deviation
Initial set of points (200 random points)	1.66	±0.05	200 random points set	1.66	±0.05
Mirror reflection (400 points)	1.64	±0.05	400 random points set	1.66	±0.04
Point reflection (400 points)	1.66	±0.06			
Six-fold rotational symmetry (1200 points)	1.65	±0.07	1200 random points set	1.68	±0.02
Initial set of points (1000 random points)	1.68	±0.02	1000 random points set	1.68	±0.02
Mirror reflection (2000 points)	1.68	±0.01	2000 random points set	1.67	±0.01
Point reflection (2000 points)	1.67	±0.02			
Six-fold rotational symmetry (6000 points)	1.67	±0.02	6000 random points set	1.68	±0.01
